# Early use of endotoxin absorption by oXiris in abdominal septic shock: A case report

**DOI:** 10.1097/MD.0000000000019632

**Published:** 2020-07-10

**Authors:** Tiantian Wei, Zhiwen Chen, Peiyun Li, Xin Tang, Mark R. Marshall, Ling Zhang, Ping Fu

**Affiliations:** a Department of Nephrology, West China Hospital, Sichuan University, 37 Guo Xue Xiang, Chengdu, Sichuan, 610041, China; b Department of Renal Medicine, Middlemore Hospital, Auckland 93311, New Zealand; c Medical Affairs, Baxter Healthcare (Asia) Pte Ltd., 189720, Singapore.

**Keywords:** endotoxin absorption, oXiris, renal replacement therapy, septic shock

## Abstract

**Rationale:**

Septic shock leads to multiple organ failure and increases mortality rate. We reported a critical patient with abdominal septic shock, which was the first case successfully treated with continuous renal replacement therapy (CRRT) and a newly designed endotoxin removal device oXiris in mainland China.

**Patient concerns:**

A 51-year-old man developed gastric ulcer perforation after resection of a benign peritoneal tumor and had a second abdominal surgery. His blood pressure decreased to 70/40 mm Hg with oliguria, requiring large doses of noradrenaline and intravenous fluid for resuscitation. The abdominal cavity was not sutured after the second open surgery due to severe abdominal infection and distention. His leukocyte count was over 30109/L, while the blood lactic acid was 12.5 mmol/L and procalcitonin (PCT) was >100 ng/mL.

**Diagnosis:**

Since the bacterial culture of peritoneal exudate showed positive with *Enterobacter aerogenes* and *Pseudomonas aeruginosa* after the second surgery, and the patient had severe low blood pressure, hyoxemia and oliguria, combined with the laboratory tests results, he was diagnosed with Gram-negative related septic shock, acute kidney injury, and multiple organ dysfunction.

**Interventions:**

CRRT with oXiris membrane was performed for 80hours and followed by AN69 ST membranes during the subsequent 27 days. Antibiotics together with other medical treatment were applied to the patient in the meantime.

**Outcomes:**

At the end of 80 hours treatment with oXiris, PCT of the patient had decreased to 14.52 ng/mL and lactic acid decreased to 4.2 mmol/L. The total sequential organ failure assessment (SOFA) score decreased from 15 to 11. Urine output steadily increased to 250 mL/h, and vital signs and blood pressure were stable without noradrenaline. At the end of the 27 days of conventional CRRT, his kidney function had completely recovered with a total sequential organ failure assessment score (SOFA score) of 6.

**Lessons:**

oXiris, with its enhanced endotoxin adsorption, appeared to accelerate improvement in organ dysfunction and ultimate survival in our patient. In critical patients with abdominal septic shock, oXiris is an important adjunctive consideration to supplement definitive source control and antimicrobial therapy.

## 1 Introduction

Septic shock and severe sepsis result in multiple organ failure and a high mortality rate.^[1–4]^ Endotoxin, which is found in the outer membrane of Gram-negative bacteria, plays an important role in the pathogenesis of septic shock by producing proinflammatory cytokines. The removal of endotoxin is a longstanding accepted therapeutic target to improve the prognosis of patients with septic shock.^[5,6]^

In recent years, continuous renal replacement therapy (CRRT) has been used for patients with severe sepsis or septic shock, both for those with and without acute kidney injury.^[7–10]^ Some CRRT devices have the common property of removing inflammatory mediators and bacterial toxins from circulation, with a view to modulating inflammatory responses and thereby alleviate end-organ damage.^[10,11]^

The oXiris device (Baxter (China) Investment Co., Ltd., Shanghai) is a CRRT filter with a membrane which has 3 layers of AN69 (polyacrylonitrile), coated on the blood side with the polycationic polymer polyethyleneimine (PEI), and subsequently saturated with unfractionated heparin (3000 UI/m) during the manufacturing process to alleviate pro-thrombogenic properties. In this device, the PEI can selectively absorb endotoxin to subsequently reduce cytokine levels, while the AN69 matrix can adsorb cytokines directly.^[11–13]^ Therefore, oXiris not only has blood purification function like AN69ST membrane, but also has the ability to reduce cytokines, endotoxin, and other inflammatory mediators. However, the safety and efficacy has not been available for trials or clinical use in septic shock in mainland China.

In this study, we report the first clinical experience with oXiris in mainland China for a critical patient with abdominal septic shock.

## 2 Methods

We analyzed the clinical data of the case and discussed oXiris and other blood purification devices which could be used in septic shock patients. This study was approved by ethics committee of West China Hospital, Sichuan University. Written informed consent was obtained from the patient and the patient's family for publication of this case report and accompanying images.

### 2.1 Case presentation

A 51-year-old man was admitted to hospital because of abdominal mass and pain in September, 2017. He had a past history of a left nephrectomy and splenectomy due to traffic accident 19 years ago, but no other major health problems. For 1 year prior to admission, he felt persistent initially mild and then severe epigastric discomfort. He was subsequently scheduled for surgery after the CT scan demonstrating an abdominal tumor.

After administering a series of preoperative examinations to exclude surgical contraindication, an exploratory laparotomy, abdominal tumor resection, adhesiolysis, intestinal resection, and end-to-end bowel anastomosis surgery were performed. Pathology of the abdominal mass was of an ectopic spleen. Two days after the first surgery, developed acrocyanosis and hypotension (70/40 mm Hg) suddenly occurred to the patient. His arterial oxygen pressure dropped to 55 mm Hg and the blood hemoglobin was 69 g/L. Other laboratory test results were listed in Table 1. The patient was diagnosed with septic shock and multiple organ dysfunction (MODS).

**Table 1:**
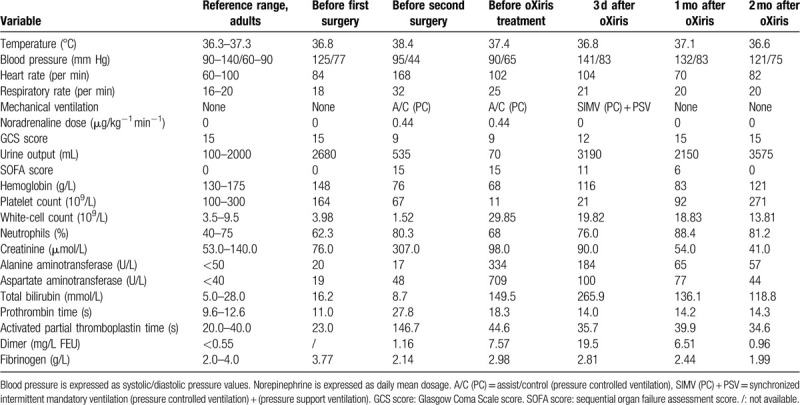
Biochemical and physiologic variables observed in the patient.

**Figure 1:**
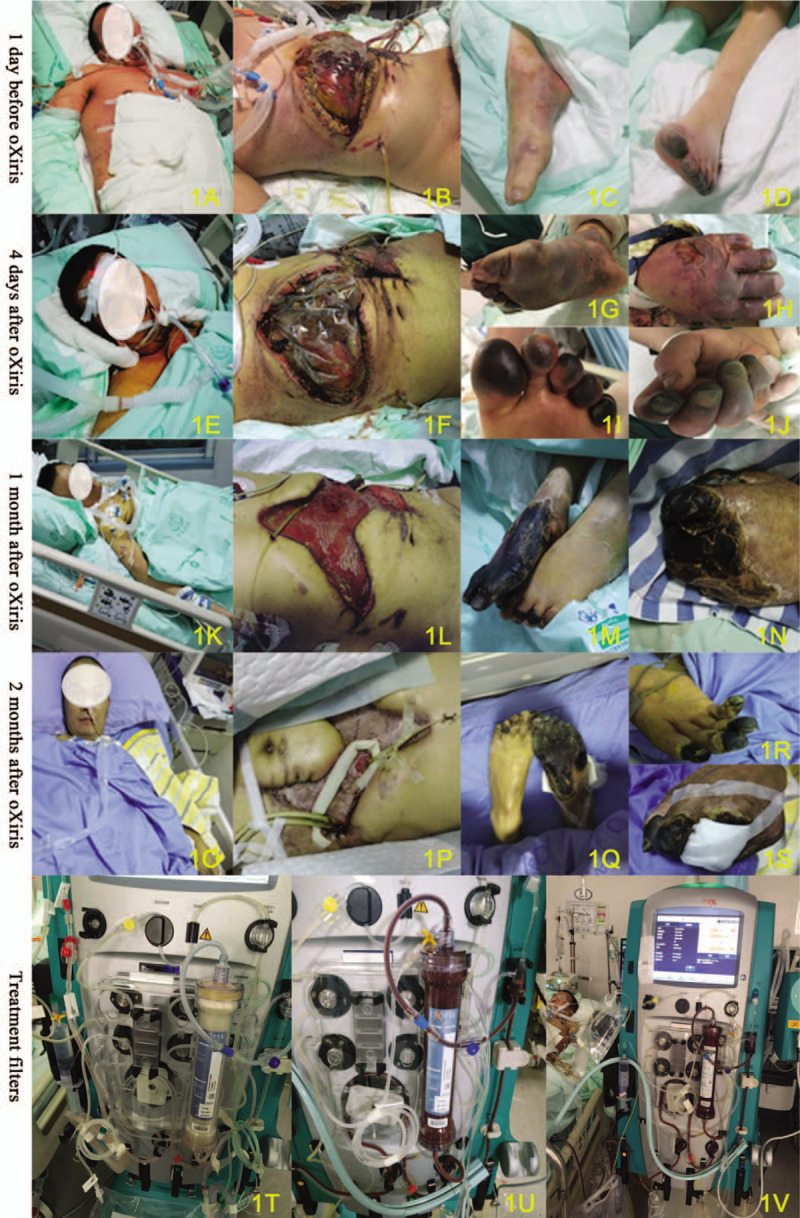
Pictures of the patient. (A–S) Pictures of the patient (A, E, K, O), his abdomen (B, F, L, P) and limbs. (A–D) 1 d before oXiris treatment; (E–J) 4 d after oXiris treatment; (K–N) 1 mo after oXiris treatment; (O–S) 2 mo after Oxiris treatment. (T–V) Pictures of the oXiris membrane (T and U) and ST150 membrane (V) during treatment.

The secondary operation was performed and a perforated ulcer on the anterior gastric wall of the patient was detected. His peritoneum was found to be grossly edematous with frank free pus. A partial gastrectomy and omental resection were applied in the second surgery. The patient's abdominal incision was not sutured because of high intra-abdominal pressure, which allowed the egress of copious purulent sanguineous discharge (Fig. 1A–D). After the second operation, the condition of the patient was very poor. The patient had severe anasarca after requiring more then 12 L of fluid resuscitation, and was hypotensive despite large doses of noradrenaline (1.50 μg/kg^−1^ min^−1^). He continued to be acrocyanotic with cold limbs, and oliguria with urine output of only 5 to 10 mL per hour. Laboratory tests results after this second surgery are listed in Table 1. Of note, the procalcitonin (PCT) concentration was over 100 ng/mL and bacterial culture test of peritoneal exudate showed *Enterobacter aerogenes* and *Pseudomonas aeruginosa*.

Antimicrobial therapy with intravenous teicoplanin, amikacin, and caspofungin as well as intravenous fluids and blood transfusion were also administered. After the surgery and medical treatment, he was still unconscious and anuria with unstable vital signs. Then CRRT was commenced with oXiris device on a Prismaflex 8.0 (Baxter, Deerfield, IL) machine for kidney support and absorption of inflammatory mediators (Fig. 1T and U). The prescription was set up as post-dilution, continuous veno-venous hemodialysis filtration (CVVHDF) with the dose of 30 mL/kg/h, and no anticoagulation was applied. oXiris filters were changed every 12 hours to insure the endotoxin adsorption efficiency.

**Figure 2:**
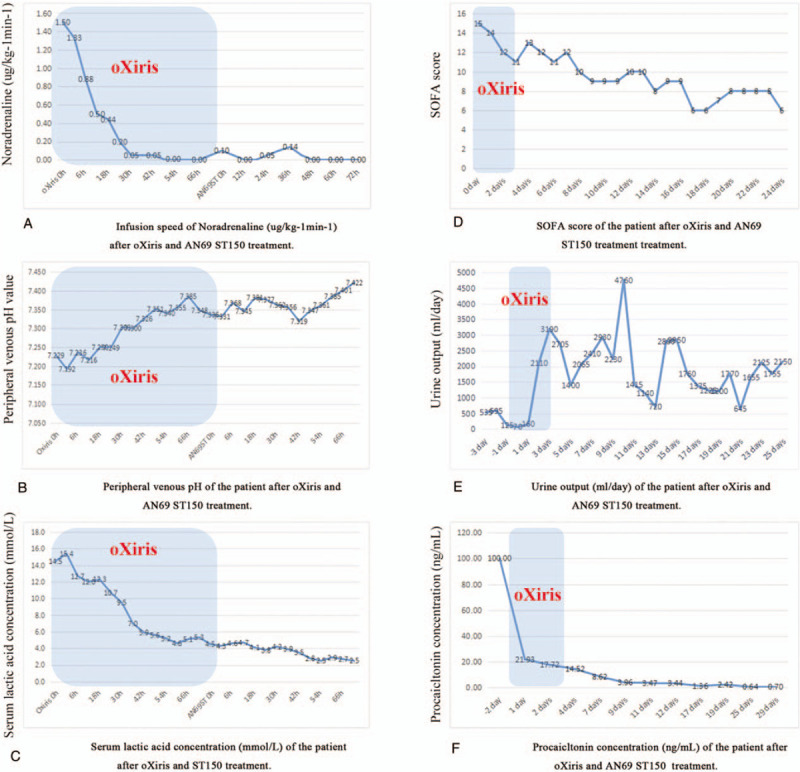
(A) Infusion speed of noradrenaline (μg/kg^−1^ min^−1^) after oXiris and AN69 ST150 treatment. oXiris 0 h: 0 h after CRRT with oXiris application; AN69 ST 0 h: 0 h after CRRT with AN69 ST150 application. Blue block: duration time of oXiris. (B) Peripheral venous pH of the patient after oXiris and AN69 ST150 treatment. oXiris 0 h: 0 h after CRRT with oXiris application; AN69 ST 0 h: 0 h after CRRT with AN69 ST150 application. Blue block: duration time of oXiris. (C) Serum lactic acid concentration (mmol/L) of the patient after oXiris and ST150 treatment. oXiris 0 h: 0 h after CRRT with oXiris application; AN69 ST 0 h: 0 h after CRRT with AN69 ST150 application. Blue block: duration time of oXiris. (D) SOFA score of the patient after oXiris and AN69 ST150 treatment treatment. 0 d: 0 d after CRRT with oXiris application. Blue block: duration time of oXiris. (E) Urine output (mL/d) of the patient after oXiris and AN69 ST150 treatment. −3 d: 3 d before CRRT with oXiris application. Blue block: duration time of oXiris. (F) Procalcitonin concentration (ng/mL) of the patient after oXiris and AN69 ST150 treatment. −2 d: 2 d before CRRT with oXiris application. Blue block: duration time of oXiris.

After 80 hours treatment with oXiris, the patient's vital signs had been stabilized and infection was well controlled. He could open his eyes and cooperate with simple physical examination (Fig. 1E). The dose of noradrenaline was progressively reduced and finally ceased (Table 1 and Fig. 2A). Sequential organ failure assessment score (SOFA score) decreased from 15 to 11 (Fig. 2D). His urine output was gradually increased from 125 to 3095 mL per day (maximum 250 mL/h, Fig. 2E). The severe disturbance in microcirculation was improved and lactic acidosis gradually abated (Fig. 2B and C). Inflammation related parameters such as PCT concentration decreased from over 100 to 14.5 ng/mL (Fig. 2F) over the 80 hours treatment period.

As the patient still had anasarca, CRRT was continued with AN69 membranes (ST150, Baxter (China) Investment Co., Ltd., Shanghai), with otherwise unchanged CRRT operating protocol. Hemofilters were changed every 24 hours (Fig. 1V). Ultrafiltrate rate was regulated according to the fluid overload. On the 27th day of admission, CRRT was able to be withdrawn after recovery of renal function, and his microcirculatory status had improved with a serum lactate of 0.6 mmol/L.

Surgical management of the patient's abdominal cavity was complicated. In addition, he developed several fistulas on his exposed intestine, which required somatostatin administration and local occlusive measurement to reduce intestinal fluid losses. Persistent peritoneal lavage and drainage was performed. Forty days after his second surgery, a right anterolateral thigh flap was transferred to cover the granulation wound of the anterior abdominal wall (Fig. 1L and P). Surgeons closed all remaining open sections of his abdominal incision in the rehabilitation phase.

This patient had several complications in this early phase in addition to AKI and local wound issues such as limbs gangrene. Within a week after the second surgery, his hands and feet were swollen and dark. Vascular ultrasound of the limbs showed that thromboembolism occluded the right dorsalis pedis artery and both posterior tibial veins. Low molecular heparin sodium and Erigeron breviscapus were applied and general improvement occurred in the conditions of his hands and feet over the next month (Fig. 1M, N, Q–S). After total of 71 days ICU treatment, MODS caused by septic shock was ameliorated. Then the patient was discharged from ICU and transferred to the general ward (Table 1).

## 3 Discussion and literature review

We present a case of abdominal septic shock, this patient was treated with CRRT and endotoxin absorption by oXiris. As per our best knowledge, it is the first report showing the oXiris device for this indication in mainland China.

Sepsis, which is caused by dysregulated host response to infection, is defined as a life-threatening organ dysfunction in Sepsis-3. As a subset of sepsis, septic shock leads to circulatory and cellular/metabolic abnormalities and substantially increases mortality.^[1–3]^ The consensus management of sepsis and septic shock is conspicuously silent on the many extracorporeal blood purification techniques, despite favorable results have been published about them in clinical trials.^[14,15]^ Removal of inflammatory mediators from circulation can modulate inflammatory responses and alleviate organ damage. Several theories espouse the potential benefit of blood purification therapy in septic patients. The “Peak concentration theory” by Ronco et al^[9]^ is the concept of eliminating peaks in soluble pro- and anti-inflammatory mediators blood concentration in septic syndromes. The “Threshold immunomodulation theory” by Honoré and Matson^[16]^ has as a core tenet that there are a minimal number of inflammatory signal pathways which are required to be controlled, the more pathways controlled the better for overall regulation of septic syndrome. Blood purification devices non-specifically remove a wide spectrum of inflammatory mediators from blood, therefore they can significantly affect tissue inflammatory activity. All of these theories highlight the importance of endotoxins and cytokines dysregulation, and the opportunity that lies in correcting the host inflammatory response. The most common therapies applied for this purpose are non- and semi-selective techniques that can remove endotoxins or cytokines (or both), such as high-volume hemofiltration, high cutoff hemofiltration/hemodialysis and high-adsorption hemofiltration.^[9]^

Of these variants, the most promising ones involve different types of CRRT membranes rather than high volume hemofiltration.^[8,9,17–19]^ High cutoff (HCO) membranes have larger-than-usual pore size (i.e., >0.01 μm), which allows the diffusion removal of middle molecular substances such as TNF-α, IL-6, IL-8, and other cytokines from serum. The use of high cutoff membrane (HCO membranes) in patients with septic AKI was initially reported as having helpful effects by reducing SOFA scores.^[20–23]^ However, the recent High Cut-Off Sepsis study (HICOSS) on patients with septic AKI showed no benefit over conventional treatment with regard to 28-day mortality, vasopressor need, duration of mechanical ventilation, or length of ICU stay.^[24]^ It is recognized that there are potential risks to HCO membranes, related to loss of valuable substances such as albumin, coagulation factors, vitamins, trace nutrients, and antibiotics. Further data are needed before definitive recommendations can be made on the use of HCO membranes in sepsis or septic AKI patients.^[25,26]^

Meanwhile, direct hemoperfusion therapy using the polymyxin B-immobilized fiber cartridge (PMX-DHP) is widely performed to treat sepsis and septic shock in Japan and Europe.^[7,27]^ Several studies on endotoxic shock reported that hemoperfusion over immobilized polymyxin-B could remove endotoxin from the circulation.^[28–31]^ There is still equipoise in the literature as to whether this technique reduces mortality in patients with severe sepsis, or even in disease-specific subgroups.^[31,32]^ The recent EUPHRATES trial has not clarified matters, other than to suggest benefit in those with higher illness severity.^[33,34]^ As with HCO membranes, it is recognized that PMX-DHP has some potential risks, and in this case these risks related to adverse events including thrombocytopenia, transient hypotension, and allergic reactions.^[35]^ In developing countries, the high price of PMX-DHP limits its usage, and for this and reasons as unproven safety and efficacy the membrane is not available in China.

oXiris (Baxter (China) Investment Co., Ltd., Shanghai) is designed to perform treatment of CRRT combined with selective adsorption of both endotoxins and cytokines, as well as renal replacement therapy in patients with sepsis or septic AKI.^[36]^ In recent years, several studies have proved that the oXiris membrane is a “broad-spectrum” device when compared with other adsorption device and can effectively eliminate both endotoxins and cytokines from blood.^[10,36]^ Clinical trails confirm that oXiris therapy is associated with an increase in blood pressure, a reduction of vasopressor requirements, and an improvement of organ function.^[36]^ It have been demonstrated that in sepsis/septic shock patients with AKI, CRRT with the adsorbing membrane oXiris might be safe and it could improve the cardiorenal-function and the clinical condition in European.^[37]^ Application of this membrane requires nothing different from conventional CRRT other than using the oXiris cartridge, thus it is simple and convenient to be applied. Meanwhile, the cost of oXiris is much lower than PMX-DHP, which makes it more affordable for patients.^[38]^ Nevertheless, the clinical experience of using oXiris is still limited in China.

In this study, our patient had severe enteroaerogen-related septic shock and MODS after peritoneal surgery. A key aspect of the case was adequate source control, definitive surgery, and appropriate antibiotic therapy. Local infection was effectively controlled by these measures. What's more, supplementary application of CRRT with oXiris not only acts as a critical treatment to AKI, but also as an effective management to sepsis syndrome and systemic manifestations. Since the antibiotics killed bacteria and oXiris removed inflammatory mediators, together with other organ supportive therapy, the patient was able to rebuilt normal host inflammatory response, recover from his acute illness, and progress to rehabilitation care.

Initial use of oXiris seems to be promising in our study, as it is illustrating the potential benefit of endotoxin/cytokine removal in severe sepsis and offering expedited safe organ function improvement. No side effect was observed during the use of oXiris in this case. Obviously, there are some limitations to this case report. Despite oXiris being able to reduce the endotoxin and cytokine concentrations, we did not measure the concentrations of these mediators in blood or CRRT effluent. In addition, this report did not prove the causality, therefore further randomized controlled trials and more real-world evidence are needed to define the benefit of oXiris hemofilter in sepsis and septic shock.

## 4 Conclusion

To our knowledge, this is the first report showing the use of oXiris hemofilter with enhanced endotoxin/cytokine adsorption capacity for septic shock patient in mainland China. Future research should investigate the benefits of early use of oXiris for severe sepsis and septic shock, confirm that CRRT with oXiris improve the recovery of MODS and other clinically relevant outcomes.

## Author contributions

**Conceptualization:** Tiantian Wei, Ling Zhang, Ping Fu.

**Data curation:** Tiantian Wei, Zhiwen Chen, Peiyun Li, Xin Tang.

**Formal analysis:** Tiantian Wei, Zhiwen Chen, Peiyun Li, Xin Tang.

**Methodology:** Zhiwen Chen, Mark R. Marshall, Ling Zhang.

**Resources:** Ling Zhang, Ping Fu.

**Supervision:** Ling Zhang, Ping Fu.

**Validation:** Ling Zhang.

**Writing – original draft:** Tiantian Wei, Mark R. Marshall.

**Writing – review & editing:** Ling Zhang.
